# Multi‐Objective Optimization of a Hydro‐Economic Model in an Over‐Allocated Agricultural Basin

**DOI:** 10.1111/gwat.70051

**Published:** 2026-03-19

**Authors:** Katherine H. Markovich, Michael N. Fienen, Nicholas Corson‐Dosch, Cecile Coulon, Jeremy T. White, Stephen B. Gingerich

**Affiliations:** ^1^ U.S. Geological Survey Upper Midwest Water Science Center Madison Wisconsin; ^2^ Present address: Department of Earth and Planetary Sciences The University of New Mexico Albuquerque New Mexico; ^3^ INTERA Incorporated Paris France; ^4^ Present address: Bureau de Recherches Géologiques et Minières Orléans France; ^5^ INTERA Incorporated Fort Collins Colorado; ^6^ U.S. Geological Survey Oregon Water Science Center Portland Oregon

## Abstract

Groundwater depletion for agricultural irrigation poses significant environmental and economic challenges. This study introduces a proof‐of‐concept that combines hydro‐economic modeling, scenario‐based modeling, and multi‐objective optimization to manage pumping curtailment in an over‐allocated basin in the western United States. Three optimization scenarios were evaluated, each offering different degrees of management flexibility. Results reveal that scenarios with finer spatial resolution achieved greater environmental benefits per unit profit loss. Additionally, strategies allowing fractional reductions in curtailed wells–rather than complete shutdowns based on water rights seniority–substantially improved efficiency, highlighting the value of increased decision‐making flexibility. Although scenario testing can aid stakeholder engagement and strategy exploration, multi‐objective optimization provides a systematic framework to quantify tradeoffs between competing objectives. This combined approach demonstrates promise for building consensus and supporting the design of sustainable water management strategies that balance agricultural livelihoods with ecosystem preservation.

## Introduction

Over‐allocation of groundwater resources for use in irrigated agriculture is a global issue, with well‐documented effects of groundwater storage loss (Wada et al. 2010; Doll et al. 2014), declining water levels (Jasechko et al. 2024), and land subsidence (Davydzenka et al. 2024). Climate change is expected to make surface water resources less reliable which could increase reliance on groundwater resources (Haile et al. 2024). If not addressed, over‐allocation can lead to profound impacts on aquatic ecosystems (Rohde, Albano, et al. 2024; Rohde, Stella, et al. 2024) and domestic well owners and farmers (Perrone and Jasechko 2017), as pumping and capital costs increase when accessing deeper groundwater levels. Although there is broad consensus that regulation of agricultural pumping in over‐allocated systems is necessary, effective implementation is challenging for complex social, political, and economic reasons (Foster and Custodio 2019; Petit et al. 2021; Hoogesteger 2022). Given the losses to livelihoods that pumping curtailment can cause, considering economically efficient solutions can aid in, maximizing environmental benefits and minimizing profit loss per gallon of pumping curtailed.

Numerical groundwater models can be effective tools to support regulatory strategy for groundwater management, as they can be used to forecast hydrologic system responses to various curtailment (or augmentation) options under a range of possible future management scenarios, while providing responses at high spatial and temporal resolutions. When paired with an agro‐economic model, so‐called hydro‐economic modeling (HEM) enables decision makers to explicitly quantify and evaluate the tradeoffs between livelihoods (expressed as profit from use of land and water) and water conservation at the field‐scale (Harou et al. 2009; Ortiz‐Partida et al. 2023). However, models are often the subject of controversy and distrust in the planning process, as they are often constructed independent of stakeholder participation (Sanz et al. 2018), and their outputs are often uncritically treated as superior to stakeholders' empirical or anecdotal experience (Petit et al. 2021).

Recognizing the need for stakeholder participation, HEM analyses can be implemented as part of the decision‐making process in a scenario‐based approach as a collection of single HEM model runs representing different management options (Jaeger et al. 2024). The advantage of scenario testing is the ability to incorporate expert knowledge and stakeholder preferences into any number of scenarios, limited only by physical representation in the model. The downside of these approaches is that there is often a range of possible combinations of decision variables that yield similar simulated outcomes for a given scenario design, even when considering the constraints imposed by existing water rights structures and regulations. Because scenario testing does not explore the full range of variability within a given management scenario, decision makers may be limited in their understanding of the sensitivity (and relative efficiency) of outcomes to degrees of freedom in favorable management solutions. Further, different combinations of management decision variables within a given scenario may yield varying degrees of efficiency. To this end, simulation‐optimization has been shown to handily exploit the potential for efficiency within a given scenario design in over‐allocated basins (Schoups et al. 2006; Maneta et al. 2009; MacEwan et al. 2017). Multi‐objective optimization is particularly well‐suited to solving complex allocation challenges given the goal of conserving water with minimal consequences to development and livelihoods (Roozbahani et al. 2015; Davijani et al. 2016; Naghdi et al. 2021). Finally, recognizing that stakeholders' preferences (and tolerance for tradeoff in those preferences) likely fall along a spectrum of optimality among those competing goals (Haden et al. 2012), multi‐objective optimization could provide an effective vehicle for consensus building in the regulatory process.

This study presents a proof‐of‐concept that merges the advantages of hydro‐economic modeling, scenario‐based modeling, and multi‐objective optimization for implementation of pumping curtailment in an over‐allocated basin in the Western United States. Three optimization scenarios are explored, comparing different pumping curtailment strategies with different management degrees of freedom. We conclude with a discussion of the real‐world advantages and disadvantages of such an approach. Although this study focuses on the specific objectives, constraints, and regulatory context of a particular basin, the approach and the insights that are generated can inform the design of curtailment strategies in other over‐allocated basins.

## Methods

### Description of the Problem

The Harney Basin is a semi‐arid basin in southeastern Oregon having a primary agricultural crop of alfalfa (hay), which is grown both to support livestock within the basin and for export (U.S. Department of Agriculture 2022). Expansion of groundwater‐fed irrigated cropland led to declining groundwater levels across the basin starting in the early 1990s (Garcia et al. 2022; Gingerich et al. 2022). The Oregon Water Resources Department (ORWD) closed the basin to any new groundwater permit applications in 2016; however, groundwater levels have continued to decline, necessitating curtailment of pumping whether by state‐mandated or voluntary measures (Garcia et al. 2022; Oregon Water Resources Department 2022). The main potential effects of declining groundwater levels include impacts to shallow domestic wells and to endemic and migratory species dependent on habitat sustained by groundwater discharge to springs, streams, and lakes in the watershed. Groundwater rights in Oregon, as in much of the Western US, follow a prior appropriations system (Oregon Water Resources Department 2018). Prior appropriation prescribes that the first person to obtain a water right is the last one to be shut off in times of water shortage. Therefore, pumping curtailment is dictated by priority dates (i.e., dates when water rights are established): if pumping is to be reduced, senior water right holders (with older priority dates) have priority over junior water right holders (with newer priority dates).

The Harney Basin was chosen as the case study for this optimization analysis given the availability of a recently published HEM (Gingerich 2024; Jaeger et al. 2024), and also because the competing objectives of economy and environment and complex constraints on solutions that the Harney Basin faces are keenly similar to many other agricultural basins in the semi‐arid western US.

### Hydro‐Economic Model

The existing HEM of the Harney Basin, developed by Jaeger et al. (2024), comprises a sequentially coupled groundwater flow model and groundwater irrigation‐economic decision model. The groundwater flow model is built in MODFLOW 6 (MF6; Hughes et al. (2017); Langevin et al. (2017)) and covers the entire Harney Basin from uplands to lowlands and simulates the period ranging from 1930 through 2018 (Gingerich et al. 2024). The groundwater flow model, described in full detail in Gingerich et al. (2024) and available at Gingerich (2024), runs at a monthly time step, simulating seasonally varying recharge, evapotranspiration (ET), and pumping as specified fluxes using the recharge and well packages, respectively, and groundwater‐fed spring‐ and base flow as head‐dependent fluxes using the drain package (Hughes et al. 2017; Langevin et al. 2017). Hydraulic properties, ET parameters, and lowland recharge locations in the MF6 model were estimated based on coherence between model outputs and groundwater‐level and streamflow measurements using a manual trial‐and‐error approach. Details of the parameter estimation performance can be found in Gingerich et al. (2024).

The MF6 model was adapted in two ways as part of the HEM development, detailed in Jaeger et al. (2024). First, the model temporal discretization was reduced down to one representative simulation year (calendar year 2018, the final year in the published groundwater flow model) of 12 monthly stress periods that is repeated to produce a 30‐year simulation period. This was done to allow for the sequential coupling of the flow model and agro‐economic decision model on an annual timescale, and the 30‐year length was chosen to reflect common planning horizons in water management. Pumping rates and locations were maintained at 2018 values for the start of each future simulation. Second, monthly recharge rates were modified to use the mean monthly rates from the period of 1982–2016 (Garcia et al. 2022). The use of a historical average rate for a 30‐year future scenario is a simplification that does not consider interannual variation, which can be substantial and could lead to differences in the relative performance of scenarios. However, because the goal of this analysis was to compare among the optimization scenarios and between the optimized results and the scenarios in Jaeger et al. (2024) and not necessarily to seek solutions that are robust to uncertainty, variability in the future scenario recharge dataset was not considered.

The irrigation‐economic decision model calculates expected profits for each of 1040 irrigated fields which are linked to one of 787 wells collocated in the same grid cell as the field (multiple fields may be irrigated by the same well). Expected profits are based on an expected price of hay, crop yield, and costs of production, including irrigation and non‐irrigation cost components.

Briefly, the profit $\pi_{\mathrm{it}}$ for a given field i in year t using irrigation technology k is:

(1) $$\pi_{\mathrm{it}}\left(k\right)={\mathrm{pY}}_{i}A_{i}-\left[C_{\mathrm{it}}^{e}\left(k\right)+C_{\mathrm{it}}^{o}\left(k\right)+C^{x}+C^{f}\right]A_{i}$$

where revenue is the price of hay (p, $US/kg) times the yield ($Y_{i}$, kg/ha) and irrigated acreage ($A_{i}$, ha), and total costs include non‐irrigation variable costs ($C^{x}$, $US/ha) and fixed costs ($C^{f}$, $US/ha), and irrigation equipment costs ($C_{i}^{e}$, $US/ha) and operating costs ($C_{i}^{o}$, $US/ha).

The expected price of hay and non‐irrigation fixed costs are held constant over the 30‐year simulation period, although irrigation technology and its associated fixed costs are allowed to change each year to maximize profit. Like recharge, the price of hay could potentially fluctuate substantially over the course of the 30‐year future scenario, and so maintaining a constant price is a simplification. Future work could explore projected price scenarios from the Global Change Analysis Model (GCAM; Calvin et al. 2019) to explore the effects of price uncertainty and variation on management optimization. The expected yield for each field was estimated using a hedonic land value regression model (described in detail in the supporting information of Jaeger et al. (2024)) and was held constant over time. The model accounts for declining groundwater levels via pumping costs (i.e., higher lift costs) and effective acreage, which is the amount of hay that can be grown given reduced well yields from declining groundwater levels and given the irrigation technology. Importantly, if a field cannot be made profitable by switching irrigation technology, it is idled in the subsequent year. Readers are referred to Jaeger et al. (2024) for more details regarding the irrigation‐economic decision modeling.

The groundwater flow and economic components of the HEM are sequentially coupled, meaning they do not interact within the groundwater model or economic model solution processes but exchange outputs and inputs at simulation timestep advancement. Following completion of the 1‐year flow simulation, the simulated depth to water for each field is transferred to the irrigation‐economic decision model, which calculates profits from the previous year and adjusts pumping rates for the subsequent year as the lesser of the available well yield or the pumping rate cap for each well. The pumping rate cap represents the maximum allowable rate for each well as prescribed by the water right. These maximum allowable groundwater pumping rates are then transferred to the groundwater flow model, which is then executed for the subsequent year using the updated allowable groundwater pumping rates.

### Management Optimization

Management optimization requires the definition of objectives, decision variables, and constraints. Management objectives represent the quantities to be maximized or minimized; constraints set the conditions that must be met by the management solutions to be considered feasible solutions, and decision variables are the adjustable parameters that the optimization algorithm modifies in its search for the best outcomes. Multi‐objective optimization maps the optimal tradeoffs among multiple, competing objectives.

#### Objectives

We defined two objectives for the multi‐objective analysis to optimize:

1. Maximize cumulative profit (π, Equation 2) across all fields i in the basin and across all years t for the 30‐year simulation period.

(2) $$\text{Maximize}\;\pi =\sum_{t=1}^{T=30}\;\sum_{i=1}^{I=1040}\pi_{\mathrm{it}}$$

**Figure 1:**
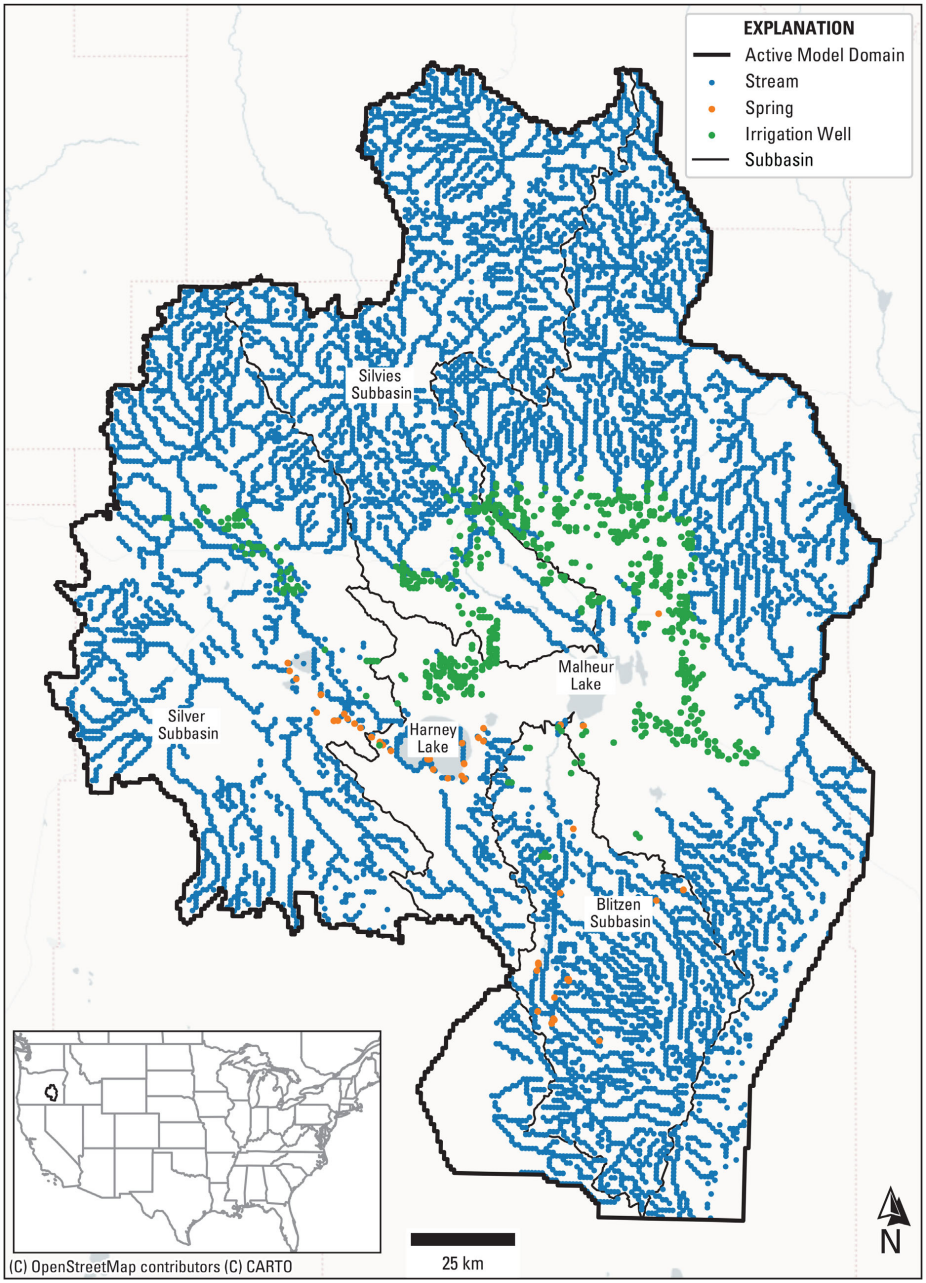
Map of active model domain (bold black outline), three major subbasins (Silver, Silvies, and Blitzen, black outline), model stream locations (blue dots), key spring locations (orange dots), and model irrigation wells (green dots).

2 Maximize cumulative volume of groundwater discharged as spring flow (V, Equation 3) across nine groups of lowland springs (j) and over the 30‐year simulation period. These groups were delineated based on clusters of mapped high discharge springs (orange points in Figure 1) that support groundwater‐dependent ecosystems (GDE). The clusters were determined manually by grouping mapped springs based on proximity and hydrogeologic characteristics. Importantly, spring response can lag changes in upgradient recharge or pumping modifications, potentially by several years (Manga 1999). Initial testing confirmed that spring discharge increased in response to year one pumping reductions by year three on average, making the 30‐year time frame more than sufficient.

(3) $$\text{Maximize}\;V=\sum_{t=1}^{T=30}\;\sum_{j=1}^{J=9}V_{\mathrm{jt}}$$

The main tradeoff present in the objectives is the desire to maximize profit (through more pumping), leading to declines in groundwater levels, versus the desire to maximize spring flow, requiring maintaining or recovering groundwater levels (through less pumping), which results in decreased profit.

#### Decision Variables

Decision variables for this work are total curtailed pumping rates and the number of years to reach those rates for groups of fields, based on three management scenarios. It is impractical both for the algorithm and in practice to adjust each field independently as a decision variable so wells must be grouped into combined decision variables. Three optimization scenarios grouping wells in different ways are considered: (1) Management areas (referred to hereinafter as the Mgmt scenario); (2) Hydrologic units (referred to hereinafter as the HUC12 scenario); and (3) Fractional reduction (referred to hereinafter as the Frac scenario). The first two scenarios represent real‐world management approaches that are constrained by prior appropriation rules; the only difference between Mgmt and HUC12 is how management areas are defined. The Frac scenario uses the same management area delineation as the Mgmt scenario, but explores an alternative approach to prior appropriation, in which wells are given fractional reductions constrained by priority date. Formulation of the decision variables differs based on scenario and are detailed below.

**Figure 2:**
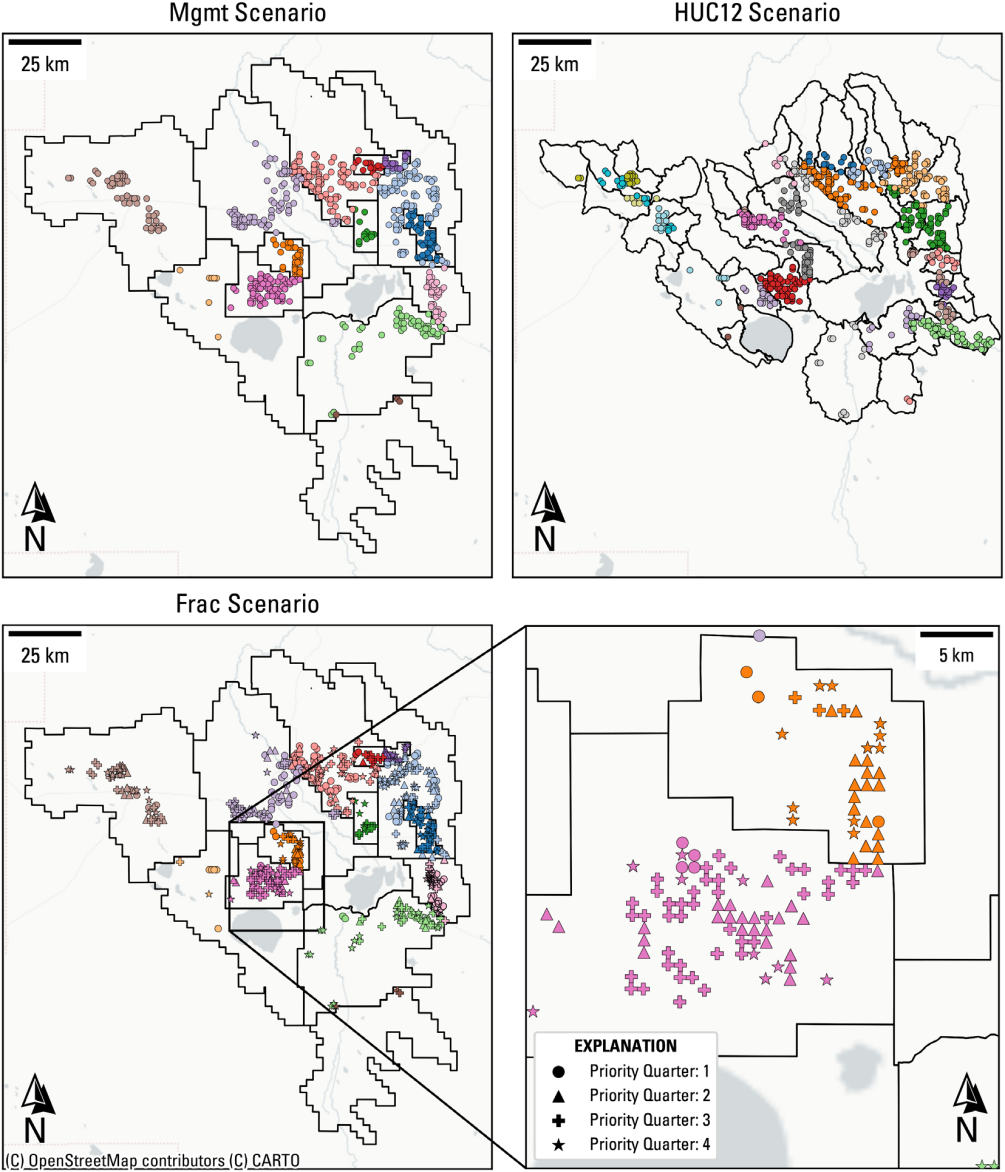
Well clusters for the three decision variable scenarios. The black polygons define scenario management areas (areas with unique decision variables). The wells are colored by their respective management area, so colors are consistent between the Mgmt and Frac scenario but change for the HUC12 scenario. For the Frac scenario, the well marker styles reflect their respective priority quarter.

For the Mgmt and HUC12 scenarios, the decision variables comprise a total curtailed pumping rate, which is the sum of pumping rates for all irrigation wells in the management area, and a number of years until the prescribed curtailed pumping rate is reached for each area, totaling two decision variables per area, where the Mgmt scenario has 14 areas and the HUC12 scenario has 38 areas (Figure 2). Together, these decision variables form a curtailment schedule, which describes how much and how quickly pumping is reduced for each management area within the model.

For management area a, the curtailment schedule $S_{a}$ ([m^3^/d]/year) is the total curtailed pumping rate for the area, $Q_{\mathrm{ca}}$ (m^3^/d), divided by the time required to reach that volume in the area, $T_{a}$ (years):

(4) $$S_{a}=\frac{Q_{\mathrm{ca}}}{T_{a}}$$

The total curtailed pumping rate for management area a can be further decomposed into:

(5) $$Q_{\mathrm{ca}}=\sum_{w=1}{{Q_{c}}_{a}}_{w}$$

where ${{Q_{c}}_{a}}_{w}$ is the pumping rate cap (or maximum allowable rate) for each well w in management area a as prescribed by the water right. For the Mgmt and HUC12 scenarios, $Q_{\mathrm{ca}}$ and $T_{a}$ from Equation 4 are defined as the optimization decision variables. The curtailment schedule is implemented in the HEM by water right priority year, in which for each management area, irrigation wells are sorted by seniority. Groundwater use allotments are cut to zero for wells starting with the most junior and moving up the chain of seniority until the total required curtailment is reached in a given year. For a longer curtailment timeline, this means that progressively more wells are cut each year, for a more gradual implementation. This use of management areas along with setting curtailment amounts and timelines to reach those amounts was designed to reflect how regulation of water rights typically proceeds in real‐world prior appropriation basins. Importantly, the sorting of wells by priority occurs within each management area independent of other management areas, so some areas may see more senior rights get cut if there are not as many junior rights within the area. This could invite dispute if the management areas are not drawn equitably (i.e., having a similar distribution of rights seniority across management areas). Barring a few areas that have just one or two wells in them, both the Mgmt and HUC12 areas encompass a reasonable spread of wells with junior and senior water rights.

The motivation for considering both the Mgmt and HUC12 scenarios is twofold: first, the HUC12 scenario contains nearly thrice as many areas for which management can be enacted, and second, the areas in the Mgmt scenario are both physical and political delineations, whereas the HUC12 is purely physical based on topography. The hypothesis being that more degrees of freedom and a more physical grouping of wells may lead to a more efficient tradeoff in the optimization (i.e., less profit loss per unit spring flow gained). Importantly, the physical and political delineations of the Mgmt scenario management areas result in 22 wells in the eastern part of the basin that are not included in the optimization and are thus not curtailed, whereas they are included in the HUC12 scenario. This could limit the Mgmt optimization by exposing slightly fewer wells to curtailment, but this was assumed to be a negligible effect compared with the difference in number of management areas between the two scenarios.

Finally, the Frac scenario decision variables comprise a percent reduction in the pumping rate cap for wells, in which wells are clustered by using the same areas as the Mgmt scenario, and further by quartiles of priority year (Figure 2). Hence, for the Frac scenario, the total pumping rate for management area a is decomposed into:

(6) $$Q_{\mathrm{ca}}=\sum_{p=1}\;\sum_{w=1}f_{\mathrm{ap}}{{Q_{c}}_{a}}_{w}$$

where p is the priority quarter, w is a well in priority group p, $f_{\mathrm{ap}}$ is the percent reduction in the pumping rate cap for the priority group, and ${{Q_{c}}_{a}}_{w}$ is the pumping rate cap (or maximum allowable rate) for each well in the group as prescribed by the water right. Thus, the Frac scenario optimization decision variables comprise $f_{\mathrm{ap}}$ from Equation 6 and $T_{a}$ from Equation 4. The priority quarters were determined based on the distribution of priority years of the irrigation wells in the model, resulting in cutoff years of 1976, 1984, and 2002. This results in five decision variables of priority‐based fractional reduction for each management area for a total of 70. The motivation for this scenario was to explore a smoother‐varying, and potentially more efficient alternative to the “on/off,” priority‐constrained process of prior appropriation. Such an approach, to the authors' knowledge, has not been applied in a real‐world system and, indeed, may not be possible in a prior‐appropriations context.

#### Constraints

Total curtailed pumping rates were constrained to vary between 50% and 100% of the 2018 pumping rates, and the number of years to full curtailment could vary from 1 to 30. The lower bound constraint for total curtailed pumping rate was chosen for fairness, as it would be unlikely that a basin management strategy would be selected such that any single management area would be allotted <50% of pumping if their neighboring areas did not also face such an extreme reduction. For the Frac scenario, bound constraints were applied to the fractional reduction decision variable based on a quartile analysis of water right priority years. The fraction was allowed to range from 1 (business‐as‐usual) to 0.6 for water rights established prior to 1976, 0.4 for water rights established from 1976 to 1984, 0.2 for water rights established from 1984 to 2002, or 0 for water rights established after 2002. Because the decision variables in the Frac scenario are unique to each priority quarter within a management area, a direct decision variable lower bound could not be set to enforce that pumping in any management area did not go below 50% of 2018 rates. Instead, this was enforced as a (nonlinear) constraint, so that solutions containing combinations of decision variables that lead to management area pumping rates below 50% of the 2018 values are treated as infeasible. This ensures that the three scenarios are directly comparable despite having different decision variable formulations.

In all three scenarios, the solution space was constrained to not retain solutions resulting in undesirable effects (i.e., drying) to springs and groundwater‐dependent streams. This involved setting a lower limit on simulated streamflow in three of the major subbasins (Silvies, Silver, and Blitzen, Figure 1), such that simulated streamflow in any subbasin does not fall below 25% of its respective simulated 2018 streamflow. Similarly, a lower limit was enforced such that volume for each spring zone did not fall below 25% of its respective 2018 flows. As curtailment may not proceed evenly across the Harney Basin in the optimization solution process, these constraints act to ensure that all solutions, at a minimum, maintain some minimal groundwater discharge to streams and springs.

### Optimization Algorithm and Workflow

Multi‐objective optimization focuses on identifying “Pareto‐optimal” solutions, or non‐dominated solutions, for which no objective can be further improved without compromising another. These solutions collectively form the Pareto frontier, which maps the optimal trade‐offs among conflicting objectives. The optimization was performed using the constrained multi‐objective optimization under uncertainty (MOU) software branch of PEST++ (PESTPP‐MOU; White et al. (2020, 2022); Welter et al. (2015)). PESTPP‐MOU contains multiple options of population generators and environmental selectors, the key components of global evolutionary algorithms (White et al. 2022). For the population generator, which generates a population of new individuals based on current individuals, this study chose particle swarm optimization (PSO) for its ability to efficiently explore a discontinuous objective function (Kennedy and Eberhart 1995; Coello et al. 2004; Siade et al. 2019). The PSO algorithm requires setting values for the social constant, cognitive constant, and inertia, which this study used 2, 0.7, and 2, respectively, to balance convergence speed with particle diversity. For the environmental selector, which selects the individuals that are “most fit” to continue into the next generation, this study uses the constrained fast non‐dominated sorting process of the Non‐dominated Sorting Genetic Algorithm‐II (NSGA‐II; Deb et al. (1995)). The population size was set to be twice the number of adjustable decision variables for each scenario, and the optimization was run for as many generations as were required for the Pareto front to converge.

Convergence was determined based on the hypervolume metric (Zitzler et al. 2003) of non‐dominated solutions and using a reference point equal to 5% greater than the maximum value of non‐dominated objective values across all three scenarios. Hypervolume is a robust metric for evaluation of multi‐objective analyses as it accounts for both optimality and diversity (i.e., “spread”) of non‐dominated solutions. If a successive generation produces solutions that dominate the previous generation's non‐dominated set, the hypervolume metric will be greater for the newer generation. This “improvement” between successive generations generally decreases as the evolutionary algorithm converges on an optimum. We therefore used a threshold criterion of hypervolume improvement to determine convergence, where the optimization scenario has converged if 10 consecutive generations show an improvement of less than half a percent. The hypervolume metric is also well‐suited as a quantitative measure of regulatory efficiency, as the scenario with the most gain in spring flow per unit of profit lost will result in the largest hypervolume, assuming the three scenarios have comparable diversity in their Pareto fronts.

For each of the three scenarios, the initial population was seeded with two explicit members (by replacing two of the stochastically generated members): a business‐as‐usual member that maintains pumping rates at their 2018 upper bound values and a maximum environmental flow member, in which decision variables related to pumping are all set to their lower bounds. This has been shown to be an effective strategy for encouraging genetic algorithms to explore the full Pareto frontier (Friedrich and Wagner 2015). Initial testing of the Frac scenario found that setting the fractional reductions to their lower bound violated the fairness constraint (not allowing any one management area pumping to drop below 50% of 2018 values) and therefore was not an effective seed for the initial population. So, to find a member that explores the spring flow maximization region of the Pareto frontier, an initial single‐objective optimization was run for the Frac scenario and the spring flow objective. This yielded a population that maximized spring flow and satisfied the bound and fairness constraints. The maximum member from this initial single‐objective optimization was seeded into the initial population for the multi‐objective optimization.

The optimization workflow is fully reproducible in python, making use of several open‐source, community‐supported software tools, including FloPy (Bakker et al. 2016; Hughes et al. 2023) and pyEMU (White et al. 2021) for building and manipulating MF6 and PEST++ analyses, respectively. The repository, including all scripts used to generate the figures for this paper, is available at https://github.com/kmarkovich/HarneyOpt and the model and PEST++ files used in generating the results shown herein are available at Markovich et al. (2026).

## Results

The hypervolume and converged generation for each optimization scenario are given in Table 1. As expected, the scenario with the fewest decision variables converged the fastest, and vice versa. Generations of 20, 25, and 40 were found to be sufficient to reach convergence for the Mgmt, HUC12, and Frac scenarios, respectively, and results are reported for those respective generations herein. Hypervolume was not only used for convergence but as a quantitative measure of optimality and, therefore, regulatory efficiency. As hypothesized, the scenario with a more flexible approach to curtailment than prior appropriation (Frac) resulted in the largest hypervolume, and the prior appropriation scenario with the fewest management areas (Mgmt) resulted in the smallest. The Frac scenario hypervolume is 23% and 15% larger than the Mgmt and HUC12 scenarios, respectively.

**Table 1:** Hypervolume and Converged Generation for Each Optimization Scenario.

Scenario	Hypervolume	Converged Generation
Mgmt	8.91E+12	20
HUC12	9.87E+12	25
Frac	1.16E+13	40

**Figure 3:**
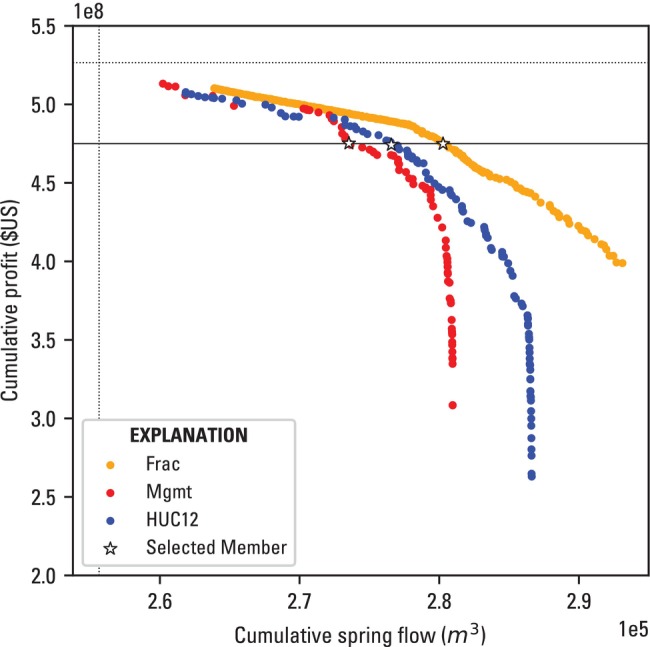
Pareto curve of feasible, optimal, and non‐dominated solutions for the three scenarios, with the solution closest to an arbitrary cumulative profit value ($4.75\times 10^{8}$ $US, black solid line) indicated by white stars. The vertical dotted line indicates cumulative spring flow and the horizontal dotted line indicates cumulative profit under business‐as‐usual (e.g., no pumping reductions) conditions.

The Pareto curves of feasible, non‐dominated solutions in the two‐dimensional solution space defined by the competing objectives for the three scenarios are shown in Figure 3. In all three scenarios, the optimization algorithm was able to find solutions that lead to large gains in spring flow with increasing cumulative spring flow, to a point, after which minimal gains in spring flow begin to occur at the cost of larger declines in profit. Notably, the vertical dotted line represents cumulative spring flow if pumping is not curtailed. The distance from this line to the solutions indicates that pumping close to “business‐as‐usual” conditions violates the minimum environmental flow constraints.

All three scenarios perform similarly in the profit maximization solution space (i.e., cumulative profit values above 5e8 $US), despite their differences in decision variable formulation, with slight efficiencies seen with the Frac and Mgmt scenario over the HUC12 scenario. As profit decreases and spring flow increases, the scenarios begin to diverge, with the Mgmt scenario having the lowest cumulative spring flow values, followed by the HUC12 and the Frac scenario, respectively.

The Mgmt and HUC12 scenarios exhibit asymptotic behavior in the spring flow maximization region of the Pareto frontier. This is due to only a handful of management areas occurring upgradient of the springs, which once curtailed to their lower limit of 50% of 2018 pumping, cease to yield any further gains in simulated spring flow. The optimization algorithm therefore explores curtailing other management areas, which lead to lower profit and no impact to spring flow. Notably, the Frac scenario does not exhibit the classic asymptotic behavior of a Pareto frontier in the spring flow maximization space (Figure 3). This likely indicates that the lower bound constraints on the fractional reduction decision variables prevented the solutions from reaching the spring flow asymptote for this optimization scenario.

**Figure 4:**
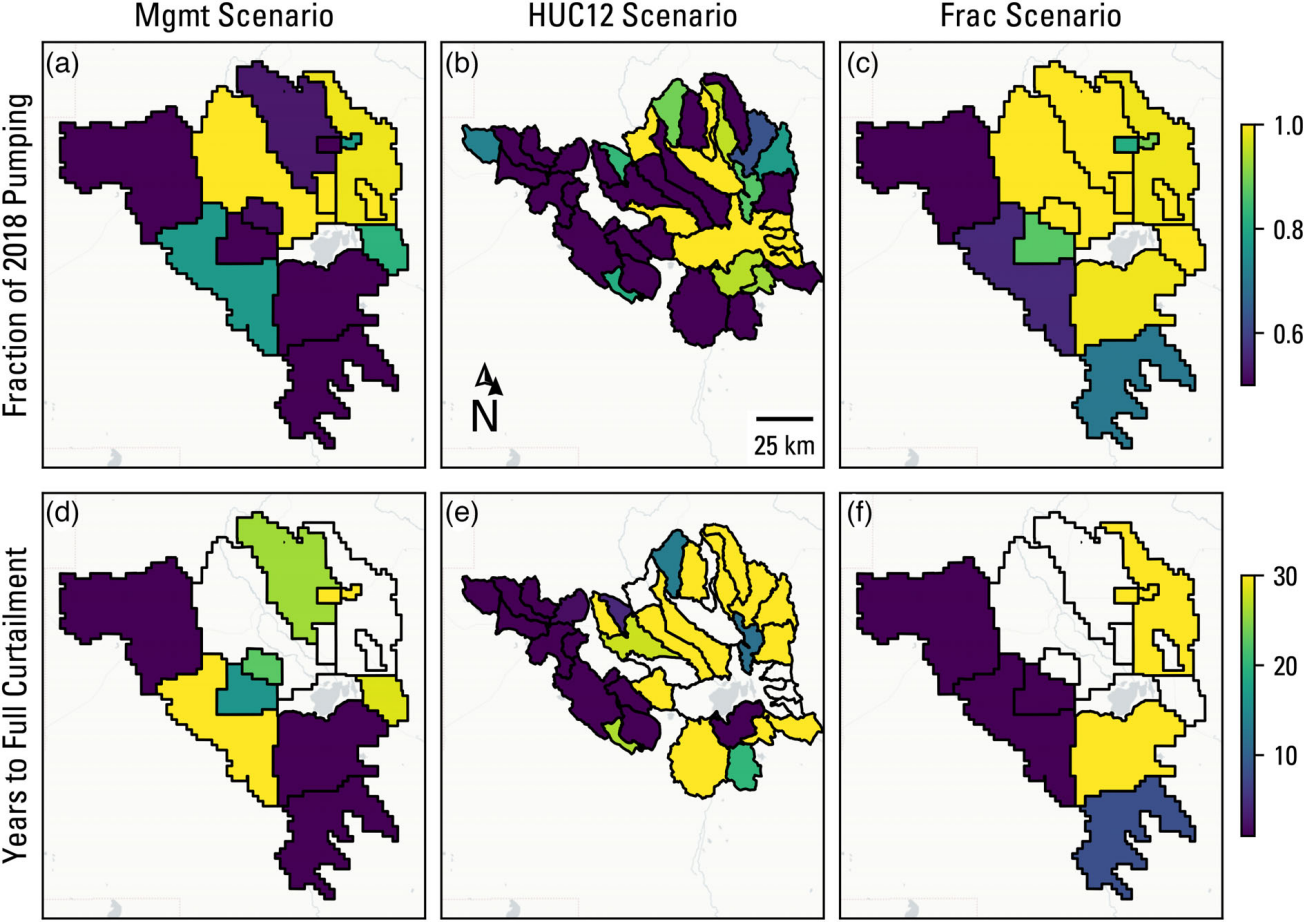
Decision variables of the member solution corresponding to the cumulative profit value of $4.75\times 10^{8}$ $US for the three optimization scenarios. The upper panel figures show the curtailed pumping as a fraction of 2018 pumping rates, and the lower panel figures show the number of years until full curtailment is reached. For management areas that were not curtailed (yellow shading in the upper panel), the respective years to full curtailment are blank.

To further explore how groundwater use is distributed in the solutions shown in Figure 3, three members from each of the optimization scenario Pareto frontier solutions were selected for comparison: a member based on an arbitrary cumulative profit value of $4.75\times 10^{8}$ $US (shown by the black outlined stars in Figure 3), a member that maximizes profit, and a member that maximizes spring flow. Figure 4 shows the decision variable values for the arbitrary profit member for all three scenarios. These three scenario solutions show a focus on maximum allowable curtailment (50% of 2018 management area pumping rates) occurring within the first year of the forecast simulation in the western and southern management areas. The preference for a short (1 year) curtailment timeline in these maximum curtailed management areas across all three scenario members reflects the cumulative spring flow objective. If the spring flow objective was formulated to be the difference in spring flow between the start and end of the forecast simulation, the optimal solutions would likely include members with longer curtailment timelines, barring any lags in spring flow response to pumping reduction.

**Figure 5:**
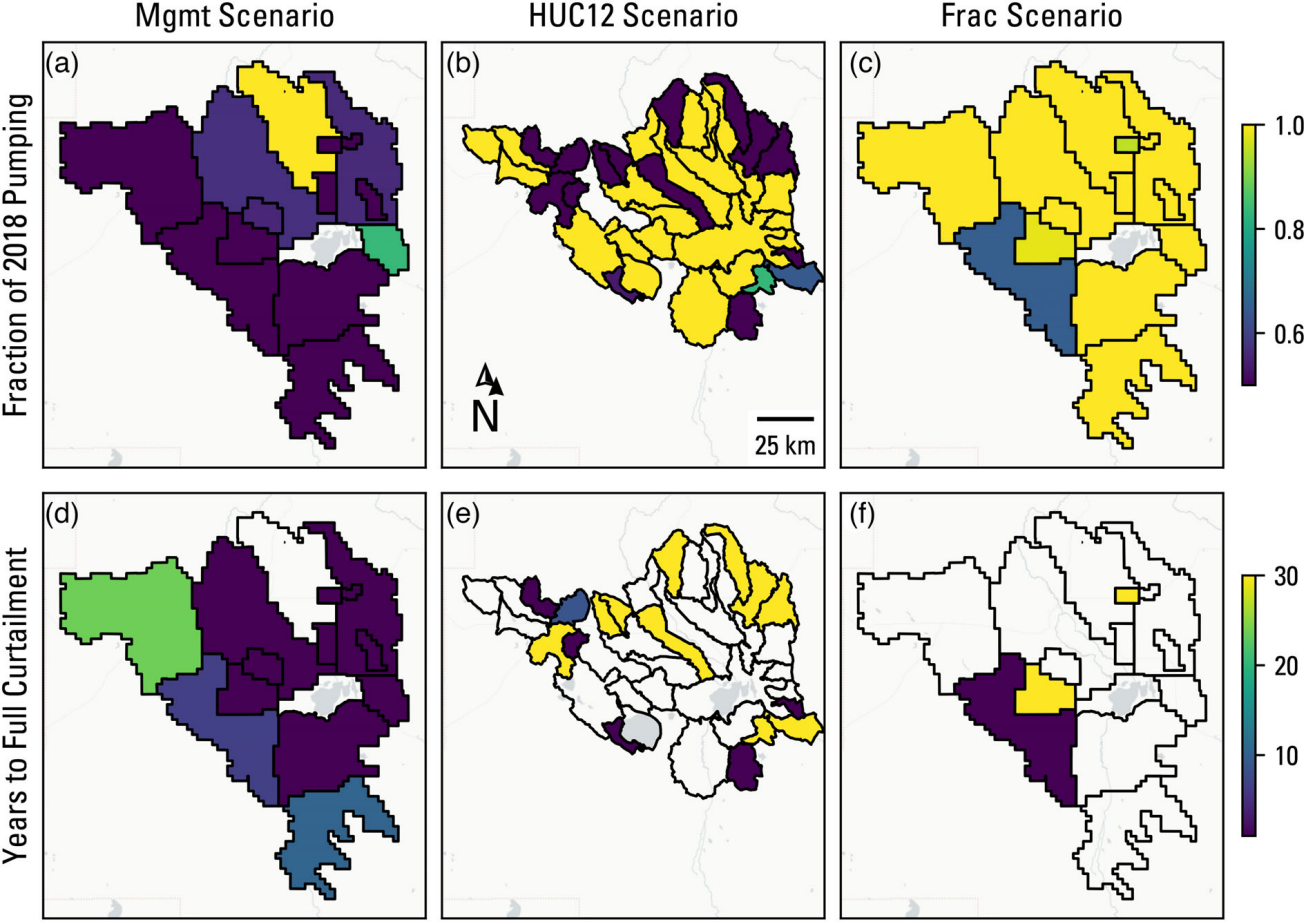
Decision variables of the member solution that maximizes cumulative profit for the three optimization scenarios. The upper panel figures show the curtailed pumping as a fraction of 2018 pumping rates, and the lower panel figures show the number of years until full curtailment is reached. For management areas that were not curtailed (yellow shading in the upper panel), the respective years to full curtailment are blank.

Figure 5 shows the decision variables for the member which maximizes profit in each optimization scenario. Note that, unlike the arbitrary profit member, the maximum profit members in the three scenarios have slightly different objective function values for profit (Figure 3). Some amount of curtailment occurs in each optimization scenario, reflecting the need to satisfy spring and streamflow constraints, much of which occurs in the western and southwestern management areas, however not to the same degree. There is much less spatial coherence than the arbitrary profit member (Figure 4), with the Mgmt scenario showing most management areas curtailed to their lower bound, whereas the HUC12 scenario member shows a handful of areas curtailed to the lower bound and the Frac scenario member shows only modest pumping reductions in a few areas. That the maximum profit member of the Mgmt scenario shows more pumping curtailment than the arbitrary member with a lower cumulative profit is seemingly counter‐intuitive but can be explained by the curtailment timeline, where the western‐most management area is not fully curtailed until 23 years in the former and immediately curtailed in the latter.

**Figure 6:**
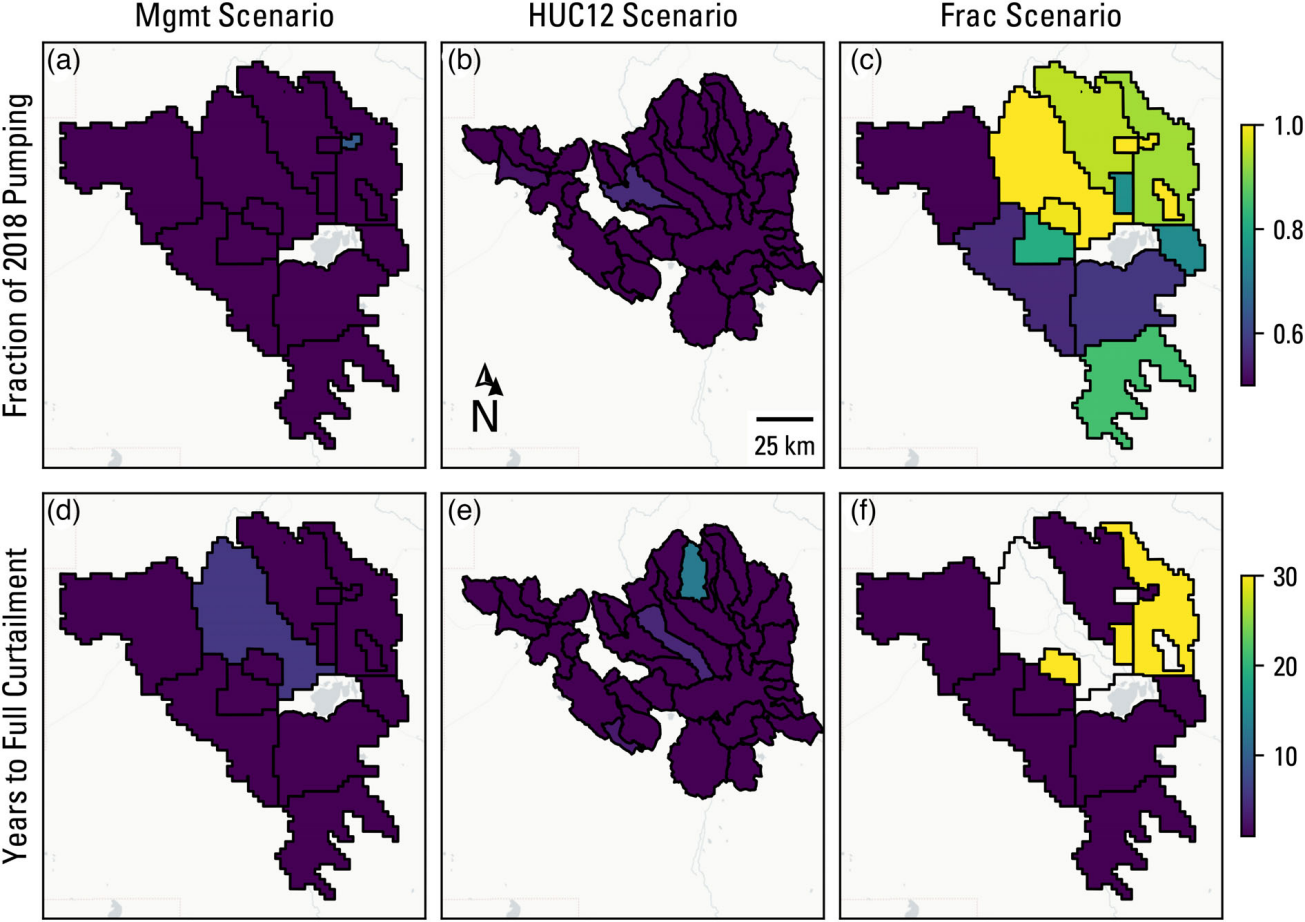
Decision variables of the member solution that maximizes cumulative spring flow for the three optimization scenarios. The upper panel figures show the curtailed pumping as a fraction of 2018 pumping rates, and the lower panel figures show the number of years until full curtailment is reached. For management areas that were not curtailed (yellow shading in the upper panel), the respective years to full curtailment are blank.

Figure 6 shows the decision variables for the member which maximizes spring flow in each optimization scenario. In the Mgmt and HUC12 scenarios, the fraction of pumping is at the 50% lower constraint in all but one management area, with years to full curtailment set to the lower bound of 1 year throughout. This is a departure from Figure 4, which resulted in a more targeted reduction in pumping, but is not unexpected for an extreme member along the Pareto frontier. The maximum spring flow member of the Frac scenario, however, shows a similar spatial distribution of decision variables when compared with Figure 4, albeit with larger reductions in pumping.

**Figure 7:**
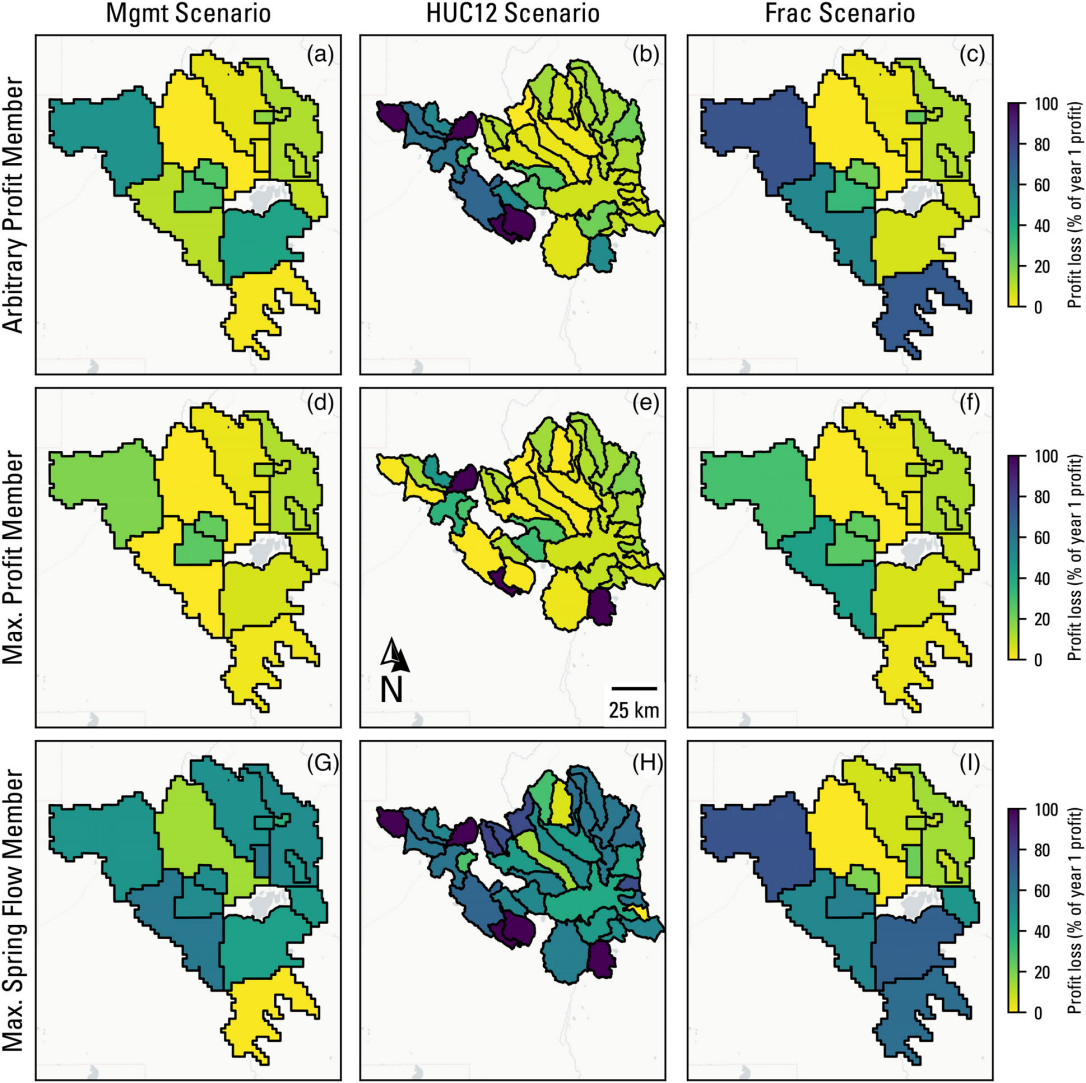
Loss in profit expressed as the fraction of profit by year 30 compared with the initial year profit in the baseline scenario by management area for selected member solutions (arbitrary, maximum profit, and maximum spring flow) from all three optimization scenarios.

Figure 7 shows management area profit loss as the ratio of profit at the end of year 30 in the three selected scenario members to the profit at the end of the first year of the business‐as‐usual run, that is, a decline in profit as a result of curtailment. Several management areas in the HUC12 scenario (areas with 100% reduction in Figure 7) show that final profit loss is 100%, which is counter‐intuitive given the 50% lower bound constraint on total curtailed pumping rate. This reflects a limitation in the decision variable approach for management areas containing few wells. If the management area has just one well in it, pumping goes to zero to reach any volume reduction. With two wells in the management area, if eliminating pumping from the first well does not reach 50%, the second is also set to zero. This points out a limitation in using a higher resolution, “physically based” management area delineation, and could be overcome by including a criterion for minimum number of wells in drawing management area boundaries.

The profit loss results (Figure 7) show reasonably good spatial coherence with the curtailment volume decision variable results (Figures 4, 5, 6), but the profit loss is regularly >50% for curtailed management areas, indicating a nonlinear relationship with the curtailment amount and timing. In other words, a 50% reduction in pumping can lead to >50% reductions in profit for a management area. This is because pumping curtailment does not guarantee a halting of water‐level decline nor uniform water‐level recovery across a management area. Continued water‐level declines from pumping lead to increased pumping costs and decreased well yields and therefore decreases in irrigated acreage and revenue. This loss in profit from declining groundwater levels is most clearly seen in the northeastern management areas that were not curtailed in any of the scenarios for the arbitrary profit member (Figure 4a–4c), yet still show a profit loss of 20% of initial values (Figure 7a–7c).

**Figure 8:**
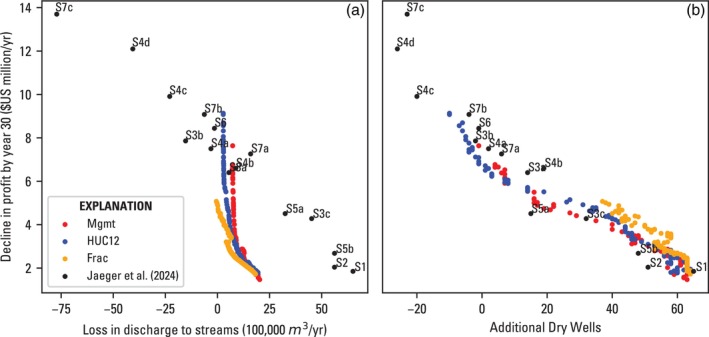
Tradeoff curve of (a) profit loss versus streamflow loss and (b) profit loss versus additional dry wells for the three optimization scenarios compared with the scenario results of Jaeger et al. (2024). Note that the optimization scenarios were not optimized for the metrics on the axes.

Finally, Figure 8 shows a comparison of the optimization scenario results to the metrics used to evaluate the 15 scenarios presented in Jaeger et al. (2024). Broadly, the 15 scenarios explored water conservation technology, land idling, pumping cost incentives, targeted regulations in areas with the largest water‐level declines, and priority‐based pumping limits. Although these are not directly comparable to the optimization scenarios in terms of decision variable formulation, all involve some form of pumping reduction. The metrics include a quantification of profit loss expressed as the difference in year 30 profit from initial profit ($17.9 M), the loss in spring discharge and stream flow by year 30 from initial values (3.21 × 10^7^ cubic meters per year [m^3^/yr]), and the gain in dry wells by year 30 compared with the initial 73 dry wells in 2018. The first two metrics are similar to the objectives of the optimization scenarios, but the profit metric is an absolute difference as opposed to a cumulative value, and the stream flow metric encompasses groundwater discharge to streams in the three major subbasins of the Harney Basin as opposed to groundwater discharge to springs clustered around the southern end of Harney and Malheur Lakes (Figure 1).

The optimization scenarios showed smaller streamflow losses (relative to initial values) per unit profit decline compared with the Jaeger et al. (2024) scenarios with profit losses <6 $US million/yr (Figure 8a). Note that the Frac solutions show discontinuous behavior near the 3 $US million/yr value, where the streamflow losses and additional dry wells shift towards the right. This indicates that the optimization found multiple configurations of management areas among which fractional reductions can achieve continuous improvements in the cumulative profit and spring flow objectives, but which produce discontinuous improvements when considering other hydrologic impacts. Towards the profit loss range of 6–10 $US million/yr, the optimization scenarios either do not overlap with or result in comparable to lower streamflow gains compared with the Jaeger et al. (2024) scenarios (Figure 8a).

For profit declines of <6 $US million/yr, the optimization scenarios resulted in a roughly $5\times 10^{5}$ m^3^/yr loss in stream discharge, and the five Jaeger et al. (2024) scenarios result in an average of $5\times 10^{6}$ m^3^/yr loss in stream discharge, an order of magnitude larger (Figure 8a). This indicates there is great potential for gains in efficiency (i.e., more streamflow is preserved per dollar unit profit decline) from using multi‐objective optimization compared with a scenario‐testing approach. However, the gains are limited by the degrees of freedom in the optimization formulation (e.g., decision variables and constraints). For example, one scenario that outperforms both the Mgmt and HUC12 optimization involved complete idling of the least profitable fields (Scenario 3b in Jaeger et al. (2024), S3b in Figure 8a). Two scenarios that reach streamflow gains (and profit losses) well beyond any of the optimization results involve increasing the cost of pumping (Scenario 4d in Jaeger et al. (2024)) and a gradual reduction in all junior pumping to 25% by the end of year 30 (Scenario 7c in Jaeger et al. (2024)).

When comparing results for the additional dry well metric, there is no clear advantage or efficiency with the multi‐objective or scenario‐based approach (Figure 8b).

## Discussion

### Regulatory Efficiency

The increase in spring flow for the same cumulative profit (Figure 3) and larger hypervolume values (Table 1) for the HUC12 versus Mgmt scenario support the hypothesis that a higher spatial resolution of management regions can lead to a more efficient solution in a strict prior appropriation setting; however, the gain (10% increase in hypervolume for the HUC12 versus Mgmt scenario) is relatively small compared with an almost tripling of the number of management areas (14–38). Ultimately, a physically based management area delineation, such as using spring “capture zones” or clustering wells based on both location and screened interval, may yield more efficient solutions. This work did not explore making each well pumping rate a unique decision variable due to the likely computationally prohibitive number of model evaluations to reach convergence. Theoretically, this would yield more efficient solutions, and further analysis could explore alternative optimization approaches that would allow for larger numbers of adjustable decision variables.

The Frac scenario exhibits the greatest gains in spring flow volumes per cumulative profit value (Figure 3) and largest hypervolume value (Table 1), indicating that degrees of freedom in terms of curtailment approach are more effective at maximizing environmental benefit per drop of water curtailed than degrees of freedom in management region delineation for the examined configuration of pumping, spring discharge, and river networks. The ability to fractionally reduce the rates of any of the wells (with priority‐based constraints on the lower limit) within a management area allows the optimization algorithm to better target the wells that are most affecting simulated spring flow. Such an approach is unrealistic given that prior appropriation is the law of the land in the case study considered (Oregon Water Resources Department 2018), but it could yield useful information for where to target land repurposing (Quandt et al. 2023) programs.

The results across all three scenarios for the arbitrary cumulative profit value of $4.75\times 10^{8}$ $US showed a high level of agreement in where pumping should be curtailed to maximize spring flow (purple management areas in a, b, and c of Figure 4), indicating that not all pumping rate curtailment is equal when seeking to find the most efficient solution. The optimization algorithm mainly targeted pumping in the higher permeability units (Gingerich et al. 2024) upgradient of and adjacent to the springs, which, intuitively, are groundwater use locations that are expected to have maximum effect on simulated spring flows. Without the 50% bound constraint, the optimization algorithm would likely curtail pumping rates even further in these same management areas. Although this would technically be the most efficient in terms of spring flow gains per basin‐wide pumping reduction, it would not be “fair” to those management areas. Fienen et al. (2017) came to a similar conclusion when optimizing pumping rate curtailment to minimize streamflow depletion. Optimization algorithms are opportunistic in their pursuit of optimality by design, so fairness should be embedded in the formulation of decision variables and constraints or objectives and weighed against losses in objective function values. The profit loss results (Figure 7) further highlight that not all pumping curtailment is equal in terms of revenue lost, because field‐level yields vary based on the hedonic land value estimation described in Jaeger et al. (2024). This underscores the value of using an HEM over a hydrologic model for this type of management optimization analysis, as relating pumping reductions to profit loss can be overly simplified in the latter approach.

### Scenario‐Based Optimization

A pure scenario‐testing approach, as in Jaeger et al. (2024), where a single model is modified and run for a range of scenarios, has a clear advantage over the optimization scenarios presented herein because it can explore a much wider range of tools and options for curtailment (Figure 8). However, there is no guarantee that any one of the scenarios is an optimal combination of the options and tools it represents. It is therefore a question of whether it can be assumed that the relative differences in a scenario‐testing approach are an accurate quantification and objective representation of the tradeoffs among them. A multi‐objective optimization approach ensures that only tradeoffs within and differences between scenarios for Pareto‐optimal members are considered (Figure 3). As demonstrated in this work, there is incredible flexibility in constraining the solution space, so no time is wasted considering options that result in undesired conditions (e.g., massive profit losses) or that are infeasible (e.g., violating spring flow requirements). Ultimately, an ideal approach could be to distill the full range of options for curtailment (such as those explored in Jaeger et al. (2024)) into a set of scenarios for optimization. This may enable a clear picture of management options and tradeoffs among them.

## Limitations

### Optimization Objectives

The spring groups were chosen as the environmental objective over maximizing stream base flow or minimizing the number of dry wells to reflect their importance for habitat for migratory and endemic species in the basin. Although maximizing all four objectives (i.e., profit, spring flow, stream base flow, and dry well count) would be ideal, limiting the optimization to two objectives greatly reduces the required number of generations for convergence. This choice of a single environmental objective was bolstered by the expectation that there would be a correlation between the three environmental quantities, and focusing on one would still benefit the others due to water‐level recovery. These results indicate that spring flow was a reasonable proxy for stream base flow (Figure 8a), but not a sufficient proxy for dry wells (Figure 8b), which is not surprising given that dry wells can occur from declining groundwater levels anywhere in the domain, whereas spring flow is only sensitive to water‐level recovery in upgradient areas that directly contribute to spring flow. Including a third dry well minimization objective would lead to tradeoffs with the profit objective and would compete with the spring flow maximization objective in terms of where curtailment is implemented. Including this objective, though requiring more model evaluations to reach convergence of the Pareto frontiers and adding complexity in the interpretation, could be an effective exercise for stakeholders during the planning phases of scenario‐based optimization (Fienen et al. 2024). This study elected to focus on the optimization sensitivity to formulation of the decision variables, but decision variable sensitivity to objective function formulation could be an area of further investigation.

### HEM Input Uncertainty

One important aspect not considered in this work is the impact of HEM model‐input uncertainty on model outputs optimization constraints. The major sources of uncertainty in the Harney Basin HEM include intrinsic properties such as aquifer hydraulic conductivity and storage and extrinsic forcings such as recharge magnitude and variability, alfalfa (hay) prices, and energy costs. These uncertainties could affect the major results of this study by reducing the number of feasible population members due to constraint violations, depending on risk tolerance (or intolerance) levels (Coulon et al. 2022). An example of this is that climatic uncertainty could lead to a small probability of the springs drying even if the upgradient management area pumping rates are cut by 50%. PESTPP‐MOU has the capability to account (and even optimize) for model input uncertainty on constraints (White et al. 2021), however, a much larger computational burden is incurred because the decision variable population has to be regularly evaluated against an uncertainty “stack.” With a one‐hour forward model runtime, this burden was insurmountable for the Harney HEM; however, future work could leverage model reduction and emulation strategies (Asher et al. 2015; MacEwan et al. 2017; Siade et al. 2020) to reduce the computational hurdle and explore scenario‐based multi‐objective optimization subject to model input uncertainty.

### Implications for over‐Allocated Basins

This work focused on using optimization to find the most efficient regulatory solutions specifically for the Harney Basin, but the competing demands and water rights system constraints are broadly applicable to other over‐allocated basins in the western US. For example, the California Sustainable Groundwater Management Act (SGMA) mandates that over‐allocated basins develop and implement plans to manage pumping to limit undesirable results such as chronic lowering of water levels, seawater intrusion, and impacts to streamflow (Harter 2020). MacEwan et al. (2017) optimized a hydroeconomic model to quantify the tradeoffs between a business‐as‐usual and managed pumping scenario for subbasins subject to the SGMA and showed that stabilization of groundwater levels actually saved farmers money in the long run when accounting for the costs of stranded wells, energy, and depleted drought reserves, a novel insight with broad implications for other basins subject to the SGMA. Their study specifically focused on subbasins where over‐exploitation has disconnected surface and groundwater (allowing for a simplified hydrologic representation in their HEM), and our results provide a case study for systems where that connection still exists. Taken together, optimization of HEMs shows potential to support decision making in these contexts.

This work was made possible due to the openly available, reproducible, and well‐documented model archive of the Harney HEM (Gingerich 2024). That work was subject to the Federal Open Government Data Act (USC 44 Section 101) which requires all Federal models to be available free to the public. Many private models are not subject to this transparency; however, this can limit opportunities for the kind of follow‐on analysis documented in this work.

## Conclusions

This work combined hydro‐economic modeling, curtailment scenarios, and multi‐objective optimization to explore the potential for regulatory efficiency in an over‐allocated basin. We showed that multi‐objective optimization was able to find a range of potential solutions even when subject to the strict implementation constraints of prior appropriation. Within a prior appropriation context, the higher the spatial resolution in management areas, the more efficiency in environmental gain per profit loss; however, the gains were not as large as anticipated in this proof‐of‐concept. A hypothetical scenario of fractional reduction (with guardrails imposed by water right priority date) produced a much larger gain in efficiency, reflecting the greater degrees of freedom in deciding which wells within a management area could be cut back. Finally, we showed that, use of scenarios is effective for exploring a wide range of curtailment approaches and for incorporating stakeholder knowledge and preferences, but multi‐objective optimization can seek optimal tradeoffs with respect to the decision objectives within any one scenario design. Provided that there is trust in and understanding of the model and optimization formulation, we posit that scenario‐based multi‐objective optimization can be used to build consensus and support strategies for sustainable management of farmer livelihoods and environmental resources.

## Data Availability

All of the workflow elements used to complete these analyses are available for download at https://github.com/kmarkovich/HarneyOpt, including the python environment, workflow script, and pre‐compiled binaries for MODFLOW 6 and PEST++. The model and PEST++ files used for generating the results shown herein are available at Markovich et al. (2026).
